# Usability of daily SF36 questionnaires to capture the QALD variation experienced after vaccination with AS03_A_-adjuvanted monovalent influenza A (H5N1) vaccine in a safety and tolerability study

**DOI:** 10.1186/s12955-019-1147-4

**Published:** 2019-05-06

**Authors:** B. Standaert, T. Dort, J. Linden, A. Madan, S. Bart, L. Chu, M. S. Hayney, M. Kosinski, R. Kroll, J. Malak, G. Meier, N. Segall, A. Schuind

**Affiliations:** 1 GSK, 20 Avenue Fleming, 1300 Wavre, Belgium; 2 Keyrus Management SA c/o GSK, Wavre, Belgium; 3 GSK, Rockville, MD USA; 4 Optimal Research LLC, Rockville, MD USA; 5 Benchmark Research, Austin, TX USA; 6 School of Pharmacy, University of Wisconsin-Madison, School of Medicine and Public Health, Madison, WI USA; 7 Optum, Lincoln, RI USA; 8 Seattle Women’s: Health, Research, Gynecology, University of Washington, Seattle, WA USA; 9 University of Wisconsin-Madison, Madison, WI USA; 10 Eisai, Woodcliff Lake, NJ USA; 11 Clinical Research Atlanta, Stockbridge, GA USA; 12 Present address: Biogen International GmbH, Baar, Switzerland

**Keywords:** Avian influenza A, H5N1, SF-36v2 questionnaire, Quality of life, Reactogenicity, Adjuvanted vaccine

## Abstract

**Background:**

This study aims to describe the short-term reactogenicity of the AS03-adjuvanted H5N1 vaccine expressed through adverse events (AEs) and quality-adjusted life-day (QALD) scores. The AEs are likely to be short-term and therefore the quality of life (QoL) questionnaire, SF-36v2, was administered daily to record changes over seven days. A more sensitive application of this instrument should allow for a better understanding of short-term tolerability of adjuvanted vaccines.

**Methods:**

Participants (*N* = 50) received a 2-dose vaccination schedule. Solicited (collected daily: days 0 to 7 [post dose 1] and 21 to 28 [post dose 2]) and unsolicited (collected weekly until day 21) AEs were collected via diary cards. The QoL questionnaires were completed daily (days 0–6) and weekly (days 0, 6, 21, 27) after dose one. Questionnaire data were transformed into SF-6D scores to report QALDs. It was hypothesized post-hoc that the QALD and daily AEs scores should correlate if discrete QoL-changes were captured.

**Results:**

Pain (92%) and muscle ache (66%) were the most commonly reported solicited local and general AEs respectively, neither increased in intensity nor in frequency after dose 2. No safety concerns were identified during the study. A correlation between the daily AEs and QALD scores existed (correlation coefficient, − 0.97 (*p* < 0.001)). The impact of the AEs scores on the QALD was marginal (− 0.02 max for one day).

**Conclusion:**

Similarly with other H5N1 studies, no safety concern was identified throughout the study. Some time-limited variations in QALD-scores were reported. Our results imply that daily administration of the SF-36v2 captures changes in QALD-scores.

**Trial registration:**

ClinicalTrials.gov. NCT01788228. Registered 11 February 2013.

**Electronic supplementary material:**

The online version of this article (10.1186/s12955-019-1147-4) contains supplementary material, which is available to authorized users.

## Introduction

In the last decade, 597 human cases of avian-origin H5N1 infections have been reported, of which 296 were fatal [1]. To date, human-to-human transmissions have been rare, however virus re-assortment or mutation could increase the potential for a pandemic outbreak. In 2016, ten years after launch, the Global Action Plan for Influenza Vaccines continued to stress the need for an increased supply and for novel innovations to enhance the preparedness of a pandemic scenario [2]. Current recombinant influenza vaccines require high antigen doses due to their poor immunogenic effects. The large amounts of antigen needed for these vaccine formulations therefore limits the global supply [3]. Adjuvantation of vaccines could overcome these issues, as adjuvanted vaccines may perform better since they have been shown to elicit a higher immune response compared with non-adjuvanted vaccines [3–5], and may in turn lead to better prevention against emerging, antigenically distinct clades of H5N1 [6], and specific infections [7]. Higher immunogenicity vaccines, however, have been shown to be associated with increased reactogenicity at the injection site (most commonly with pain and swelling) and systemic symptoms (usually with fatigue, muscle soreness and headaches) [8–10]. The potential impact on the overall quality of life (QoL) of an individual receiving an adjuvanted vaccine expressed as a measure over time in quality-adjusted life-years (QALY) or days (QALD) score loss has never been reported. Most reactogenicity occurs within a few days post-vaccination only, therefore novel and sensitive QoL instruments that capture daily changes are required.

This study assessed the reactogenicity of AS03-adjuvanted H5N1 vaccine in terms of solicited local and general adverse events (AEs), and its impact on general QoL and well-being with the use of the Short-Form-36v2 health assessment questionnaire (Additional file 1: Figure S1) [11]. Using the SF-36v2 questionnaire on a daily basis is a novel approach in collecting discreet changes in QoL within a short period that can be translated into a utility score which allows measuring QALYs or QALD. This approach requires quantitative differences between the daily and weekly questionnaire scores to be compared with base-line values and with solicited and unsolicited AE data to analyze the psychometric validity and internal consistency of the daily questionnaire after transformation into a SF-6D utility score. The SF-6D or Short form-six dimension is a method to calculate those scores that include trade-offs between different subscale components of health, for example pain and physical functioning [12].

## Methods

This was an open-label, uncontrolled, multi-center (*N* = 3) study with a total duration of approximately 385 days for each participant (ClinicalTrials.gov identifier NCT01788228). The work was conducted in the USA between 22 March 2013 and 25 February 2015 and included employees in government and non-government institutions (e.g., Centers for Disease Control and Prevention, the National Institutes of Health, the Department of Agriculture, the Food and Drug Administration, the Department of Defense, state and local government, and research universities) who were at occupational risk of H5N1 exposure and were able to participate through one of the 3 study sites (Clinical Research Atlanta, GA, USA; Accelovance, MD, USA; Benchmark Research, TX, USA and UC Davis Medical Center, CA, USA; and University of Wisconsin Health Services, WI, USA). It was anticipated that the enrollment target of 150 subjects would be completed in 10 months; however, recruitment was slower than expected due to the low number of adults at occupational risk of H5N1 virus exposure thus only 50 participants were included. This study was conducted in accordance with the Declaration of Helsinki and the Good Clinical Practice. The research and all appropriate documentation were reviewed and approved by the Independent Ethics Committee or the Institutional Review Board (Chesapeake Research Review Inc., Columbia, MD, USA and Western Institutional Review Boards, Puyllup, WA, USA). Written informed consent was obtained from each participant before the performance of any study-specific procedures.

**Fig. 1:**
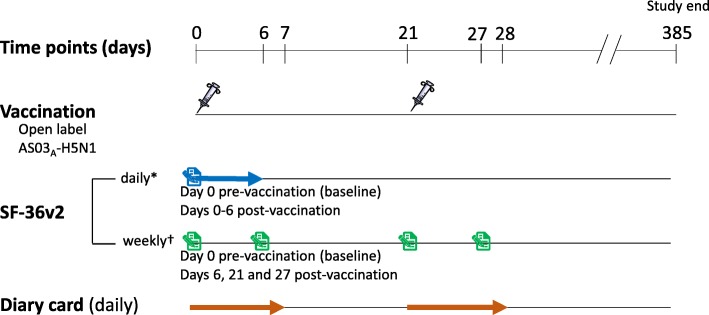
Study Design Schematic. AS03_A_-H5N1, AS03_A_-adjuvanted H5N1 vaccine; *, Questionnaire assesses health and well-being for the previous 24 h; †, Questionnaire assesses health and well-being for the previous week

The vaccine was evaluated through instruments measuring solicited local and general AEs, specifically, reactogenicity and QoL parameters. All reactogenicity data was collected, via diary cards, over the first seven days after each vaccination (Days 0 [baseline, dose 1] to 7, and 21 to 28 [dose 2]) and all unsolicited AEs for 21 days after each dose (Fig. 1). Participants were instructed to complete QoL measures weekly (Days 0, 6, 21 and 27) and daily (pre-injection on Day 0 [baseline], and post injection on Days 0 to 6) which would represent a more discreet time-interval (Fig. 1). Daily questionnaires were not used after the second dose as a broad (approximately 7000 participants) clinical study had already established that solicited local and general AEs following the second injection are very similar to those following the first dose [8]. Our daily questionnaire was used as a novel approach to assess the potential sensitivity (0 to 6 days) of a generic QoL instrument and not to re-examine the results of the sizeable H5N1 study. The adverse pregnancy outcome and medically attended adverse events (MAEs), including potential immune-mediated diseases (pIMDs) and serious adverse events (SAEs), were also recorded to describe the long-term safety (385 days after the first vaccination).

### Study vaccine

All participants received two doses (vaccinated on Days 0 and 21) of Influenza A (H5N1) Virus Monovalent Vaccine Adjuvanted. The first treatment was administered in the deltoid region of the non-dominant arm and the second dose in the deltoid region of the dominant arm. Each dose consisted of an antigen and adjuvant mix (1:1) and included 0.25 mL A/Indonesia/5/2005 antigen (3.75 μg per dose) and 0.25 mL of AS03_A_ (Adjuvant System containing α-Tocopherol [11.86 mg] and squalene in an oil/water emulsion). Each 0.5 mL dose of vaccine delivered approximately 5 μg thimerosal. The vaccine was developed and manufactured by GSK.

### Study population

Study participant inclusion were those considered at risk of occupational exposure to H5N1 influenza virus including males and females ≥18 years of age at the time of the first vaccination and who had demonstrated stable health 30 days before enrollment. Female participants of childbearing age had negative pregnancy tests and had been practicing adequate contraception at least 30 days before the first dose, and agreed to continue contraception for at least two months after the last vaccination.

The exclusion criteria included participants unable or deemed unable to provide accurate safety information due to neurological or psychiatric diagnoses. A receipt of an inactivated vaccine within 14 days, a live attenuated vaccine within 30 days of the first vaccine dose, or any vaccine not foreseen by the protocol before Day 42 would all result in exclusion from the study. Other reasons for exclusion included significant risk of complications from intramuscular injections due to a disorder of coagulation, temperature ≥ 38 °C on the day of first vaccine dose, acute evolving neurological disorder or Guillain Barré Syndrome within 42 days of receipt of prior seasonal or pandemic influenza vaccine, a history of allergy to influenza vaccine and pregnant or lactating women.

### Statistical methods

All data sets were characterized by descriptive analysis, including demographic characteristics (age at first study vaccination in years, gender, and ethnicity), vaccination history, cohort description and withdrawal status. The primary analysis of safety was performed on all study participants. All analyses used SAS 9.2 (SAS Institute, NC, USA) unless stated otherwise below. The according to protocol (ATP) cohort for analysis of safety was not performed since no vaccinated participants were eliminated from the ATP cohort for analysis of safety. Analysis by age stratification was not performed as only one participant was over the age of 64 years.

#### Safety

Safety data were analyzed by the participant incidence of solicited and unsolicited AEs, by solicited local and general symptom terms and, for unsolicited AEs, by the primary Medical Dictionary for Regulatory Activities (MedDRA) preferred term and system organ class. The incidence of solicited local and general AEs and unsolicited AEs that occurred during their respective follow-up periods (Fig. 1) were tabulated with exact 95% confidence intervals (CIs). The same calculations were performed for symptoms by grade (0–3), as well as for solicited general AEs considered to be related to vaccination. All of the solicited local AEs were considered to be causally related. The MAEs, SAEs and pregnancy were summarized over the entire period of the study.

#### Quality of life

The analysis was performed on all of the study participants. Data sets were generated using the QualityMetric Health Scoring Software Assessments for the weekly and daily QoL questionnaires. The rationale for having both weekly and daily quality of life questionnaires after the first dose was to assess and validate the daily questionnaire with respect to the psychometric validity and internal consistency of the daily SF-36v2 questionnaire. Both validity and consistency will be concurrently assessed by evaluating quantitative differences between the SF-36v2 weekly and SF36v2 daily questionnaire responses, as well as quantitative differences between SF-36v2 questionnaire responses and solicited and unsolicited AE data.

Quality Metrics performed the psychometric validity, internal validity, consistency and test/retest reliability of the daily SF-36v2 questionnaire in order to validate the daily QoL datasets. SF-6D data was generated by GSK after transformation of the SF-36v2 results to produce daily utility scores that can be presented as QALD-scores as the recall period of the QoL questionnaire has the same time unit as the utility assessment. The SF-36v2 questionnaire has been extensively validated with procedures reported in Ware et al. (2008) [13]. Briefly, delivered weekly, this generic, multi-purpose questionnaire has 36 questions generating an 8-scale profile of scores. They include physical function, physical health-related role limitations, bodily pain, general health perceptions, vitality, social function, emotional health-related role function, and mental health. These combined make up the subscale component scores. Summary component scores (categorized as physical [PCS] and mental [MCS]) and utility scores are generated based on these profile scores.

Descriptive analyses were generated for each subcomponent score, overall component score and utility scores for both the weekly and daily questionnaires. Analyses of daily questionnaire results were carried out using Stata 14 (StataCorp LP, TX, USA).

The values for the weekly questionnaires were assessed overall by the presence or absence of grade 3 solicited AEs after each dose. Descriptive statistics were also generated for changes from baseline (Day 0) for three consecutive weeks after that. Daily questionnaire values were assessed overall and for participants who reported AEs during a seven-day period after dose one. Descriptive statistics were also generated for changes from baseline on all seven days and ‘time to return to baseline’ for participants who experienced AEs in the seven days post-dose one. The time to return to baseline was calculated as follows: 1) only negative (decrease) deviations from the baseline were considered. For any variable the number of days it took to return to baseline (0), or above (> 0), were calculated but only if it went below the baseline and then returned as equal to, or above, the baseline within the seven days. 2) For any variable, if there was no variation then no value was given. 3) If there was a decrease and then a recovery as described in point 1) followed by a second decrease and recovery, then only the first period to recovery would be counted. This point is based on the assumption that the second decrease and recovery period is less likely to be associated with the vaccination as the first. The summed solicited AEs for each day (Days 0–6) were correlated with the daily average utility scores over the same period using Pearson correlation test in a post hoc analysis.

## Results

### Demographics

A total of 50 participants were enrolled before the recruitment period was terminated. No participants were excluded from the ATP for safety analysis as all participants completed the study ATP and were included in the analyses. One participant did not complete the study after relocating from the study area and only one participant was enrolled in the ≥65 age stratum. Summary demographics are presented in Table 1. A total of 96% of the participants had received a seasonal influenza vaccination before the study. Only 14% of the participants reported having received a previous H5N1 vaccination (2% did not know).

**Table 1:** Summary of demographic characteristics of the total vaccinated cohort

			N = 50
Characteristics	Parameters or Categories	Value or n	%
Age (years) at study start	Mean	43.3	–
	SD	12.2	–
	Median	41.5	–
	Minimum	26	–
	Maximum	85	–
Gender	Female	28	56
	Male	22	44
Ethnicity	American, Hispanic or Latino	4	8
	Not American, Hispanic or Latino	46	92
Geographic Ancestry	African Heritage/African American	3	6
	American Indian or Alaskan Native	0	0
	Asian – Central/South Asian Heritage	2	4
	Asian – East Asian Heritage	3	6
	Asian – Japanese Heritage	3	6
	Asian – South East Asian Heritage	3	6
	Native Hawaiian or Other Pacific Islander	0	0
	White – Arabic/North African Heritage	0	0
	White – Caucasian/European Heritage	34	68
	Other	2	4

*N* total number of participants, *n*/% number or percentage of participants in a given category, *SD* standard deviation

### Reactogenicity

#### Weekly diary cards after each dose

A total of 92% of the participants experienced local AEs over the course of the two doses and 66% of the participants reported general AEs. In total, 96% of the participants who had any AEs, only 10% were grade three (Table 2). There was no notable difference in the frequency or intensity of solicited local or general AEs after the first and second dose of vaccine; local AEs Dose 1 (D1) = 86% and D2 = 80% and general AEs both doses = 56% (Table 2).

**Table 2:** Incidence and nature of symptoms (solicited only) with causal relationship to vaccination, reported during the 7-day (Days 0–7 for dose 1, and Days 21–28 for dose 2) following each dose and overall (TVC)

	Local symptoms					General Symptoms					Any Symptoms				
				95% CI					95% CI					95% CI	
	N	n	%	LL	UL	N	n	%	LL	UL	N	n	%	LL	UL
All causally related symptoms															
Dose 1	50	43	86	73.3	94.2	50	28	56	41.3	70.0	50	45	90	78.2	96.7
Dose 2	50	40	80	66.3	90.0	50	28	56	41.3	70.0	50	46	92	80.8	97.8
Overall/dose	100	83	83	74.2	89.8	100	56	56	45.7	65.9	100	91	91	83.6	95.8
Overall/participant	50	46	92	80.8	97.8	50	33	66	51.2	78.8	50	48	96	86.3	99.5
Grade 3 causally related symptoms															
Dose 1	50	3	6	1.3	16.5	50	2	4	0.5	13.7	50	4	8	2.2	19.2
Dose 2	50	2	4	0.5	13.7	50	1	2	0.1	10.6	50	2	4	0.5	13.7
Overall/dose	100	5	5	1.6	11.3	100	3	3	0.6	8.5	100	6	6	2.2	12.6
Overall/participant	50	4	8	2.2	19.2	50	2	4	0.5	13.7	50	5	10	3.3	21.8

*TVC* total vaccinated cohort

For each dose and overall/participant: *N* number of participants with at least one documented dose, *n/%* number/percentage of participants presenting at least one type of symptom whatever the study vaccine administered

For overall/dose: *N* number of documented doses, *n/%* number/percentage of doses followed by at least one type of symptom whatever the study vaccine administered

#### Solicited local symptoms

The most frequent solicited local AE, pain, was experienced by 92% of participants and redness and swelling were reported by 12 and 14%, respectively. Grade 3 pain was reported by 8% of participants, while there was no report of either redness or swelling reaching over 100 mm (Table 3).

**Table 3:** Incidence of solicited local and general symptoms reported during the 7-day period after each dose (Days 0–7 for dose 1, and Days 21–28 for dose 2), overall by participant (TVC)

					95% CI	
Symptom	Type	N	n	%	LL	UL
Solicited local symptoms						
Pain	All	50	46	92	80.8	97.8
	Grade 3	50	4	8	2.2	19.2
Redness	All	50	6	12	4.5	24.3
	Grade 3	50	0	0	0.0	7.1
Swelling	All	50	7	14	5.8	26.7
	Grade 3	50	0	0	0.0	7.1
Solicited general symptoms						
Fatigue	All	50	27	54	39.3	68.2
	Grade 3	50	1	2	0.1	10.6
Gastrointestinal symptoms	All	50	12	24	13.1	38.2
	Grade 3	50	2	4	0.5	13.7
Headache	All	50	20	40	26.4	54.8
	Grade 3	50	2	4	0.5	13.7
Joint pain	All	50	14	28	16.2	42.5
	Grade 3	50	1	2	0.1	10.6
Muscle ache	All	50	33	66	51.2	78.8
	Grade 3	50	1	2	0.1	10.6
Shivering	All	50	10	20	10.0	33.7
	Grade 3	50	1	2	0.1	10.6
Sweating	All	50	7	14	5.8	26.7
	Grade 3	50	1	2	0.1	10.6
Temperature	All	50	1	2	0.1	10.6
	Grade 3	50	0	0	0.0	7.1
	Grade 4	50	0	0	0.0	7.1

*N* Number of participants with at least one documented dose, *TVC* Total vaccinated cohort

Symptom intensity was graded on a scale of 1 (mild) to 3 (severe) for all symptoms except for body temperature which was graded on a scale of 1 to 4. Grade 3 symptoms were defined as follows: for redness or swelling, a diameter > 100 mm; for fever, body temperature ≥ 39.0–40.0 °C (grade 4: > 40.0 °C); and for all other events, preventing normal activities

#### Solicited general symptoms

The most frequently reported solicited general AEs were muscle ache, fatigue, and headache, with overall incidences of 66, 54 and 40%, respectively (Table 3). No more than 4% of participants reported grade 3 symptoms in any specific category of solicited general AEs (Table 3).

#### Unsolicited AEs and MAEs

Ten participants (20%) reported at least one unsolicited AE up to 21 days after a vaccine dose and nine participants (18%) reported at least 1 MAE from Day 0 through Day 385. No safety concerns associated with either the unsolicited AEs or MAEs were identified. No pregnancies were reported.

### Quality of life

#### Weekly questionnaires

All participants, apart from one after the first dose, completed the weekly QoL questionnaires. However, all participants completed the weekly questionnaires after the second dose. One of the 50 participants did not complete the daily questionnaires in their entirety; two did not complete day one, one did not complete day three and one did not complete day four.

There were no noticeable changes in any of the tabulated descriptive statistics (not shown) or from baseline for any of the subscale component or component scores based on the descriptive statistics (Additional file 2: Table S1). The largest increase in mean score change was bodily pain (1.891; Standard Deviation [SD] = 7.203) on Day 21, and the largest decrease was physical health-related role limitations (− 0.837; SD = 4.467) on Day 6. Table 4 presents the average utility scores with weekly recall periods. Analysis of MAEs did not show any meaningful results, as there were too few within 21 days to make this analysis relevant. No SAEs were reported.

**Table 4:** Average utility scores with weekly recall and daily recall (= QALD)

	Weekly recall utility score	Daily recall utility score (=QALD)	Accumulated QALD-loss from baseline
Pre dose 1	0.863	0.885	
Dose 1-D0		0.874	0.011
Dose 1-D1		0.863	0.034
Dose 1-D2		0.877	0.042
Dose 1-D3		0.889	
Dose 1-D4		0.894	
Dose 1-D5		0.902	
Dose 1-D6	0.879	0.902	
Dose 2-D0	0.891		
Dose 2-D6	0.874		

*QALD* Quality adjusted life day

Dose 1 was administered on Day 0 and Dose 2 was administered on Day 21. D0 refers to the day on which vaccine was given; D6 refers to six days post dose

#### Daily questionnaires

Validity between the daily and weekly questionnaires was demonstrated, which indicates that the daily QoL questionnaire is appropriate for use.

Descriptive statistics for daily changes relative to baseline across all participants show a steady decrease in PCS from day 0 to day four and then an increase during the last two days (D5 and D6). These changes vary from − 1.858 (SD = 4.865) on day 0 to 0.599 (SD = 4.017) on day six. Changes in MCS show no general pattern and vary from day to day. Changes from baseline in utility score decrease in the first two days by − 0.0169 and − 0.0047 respectively before rising consistently over the next five days (Additional file 2: Table S2). The utility data evaluated by solicited AE status is presented in Table 4. Average daily changes relative to baseline in bodily pain were negative for day 0 and day one, but not for days 2–6. The general health perception score was negative for day 0, but not for days 1–6. The average daily change from baseline in the PCS was negative for day 0, but not for days 1–6. For the subscale component scores physical functioning, role limitations - physical health related, vitality, social functioning, and role limitations - emotional health related the average daily changes from baseline were not significantly different from day 0. The average daily changes from baseline for the summary MCS were not significantly different from day 0. There were also no significant changes from baseline (based on the daily questionnaires) for the daily utility scores. The time taken to return to baseline, or go beyond the baseline value for participants who reported an unsolicited AE in the first six days after dose one, ranged from 1.55 to 1.89 days for the subscale component scores. The summary scores values were recorded as PCS: 2.12 days, MCS: 1.89 days and daily utility scores: 1.84 days.

#### Post-hoc analysis

**Fig. 2:**
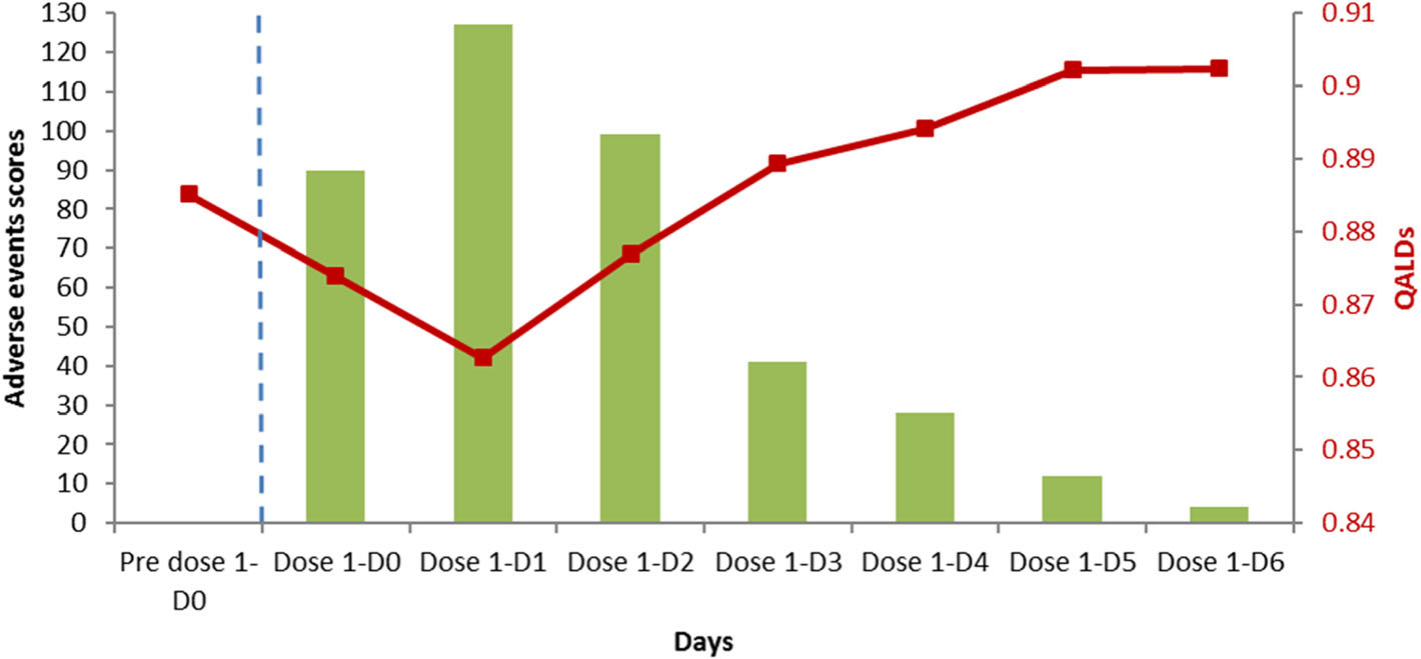
Daily sum of solicited local and general adverse events scores versus average QALD scores before and after first dose. QALD, quality-adjusted life-day. QALD data on right Y axis are also available on Table 4

A post hoc analysis was performed in which the correlation between the sum of all the solicited AEs across participants per day and the average daily utility scores of the participants who experienced solicited local and general AEs were assessed (Fig. 2). The Pearson correlation coefficient between the two scores was − 0.97661 (*p* < 0.001) indicating that a change in AE-scores per day is well captured by a change in the daily utility scores. This information is helpful to capture the utility-loss following the administration of the adjuvanted vaccine during the three days of lower utility-scores than at base-line after the injection. An accumulated estimate of − 0.042 QALD is observed which represents − 0.00011 if expressed in QALY units (Table 4).

## Discussion

This size restricted, multi-center, phase IIIb study, a 2-dose schedule of the AS03-adjuvanted H5N1 vaccine in adults aged ≥18 years who were at occupational risk to H5N1 influenza virus exposure raised no safety concerns. Nearly all participants experienced local AEs (92%) whilst two-thirds experienced general AEs. Our results were supported by the low incidence of associated unsolicited AEs up to 21 days after each administered dose and low MAEs after 385 days after dose one. The absence of any associated SAEs or pIMDs over the same period further supported this primary finding. Our results are very similar to previous large clinical studies with AS03-adjuvanted vaccines [8, 14]. Like our results, local AEs, pain and muscle ache were found to be the most common local and general AEs, reported by 89 and 45% respectively in the dose-sparing H5N1 A/Indonesia/05/2005 study [14]. Similarly, the sizeable AS03-adjuvanted influenza A(H1N1)pdm09 vaccine study by Yang et al. [8] found that pain was reported in 81% of participants, while muscle ache, fatigue and headaches were reported by 39, 31 and 30% respectively.

In this study, the SF36v2 questionnaire was also used to evaluate changes in quality of life over time and specifically to evaluate the potential of this typically weekly-administered instrument to a more sensitive period. The SF36v2 showed high potential as a sensitive instrument capable of capturing daily changes. For example, variations between daily utility/QALD scores and the sum of solicited AEs per day were apparent and we report a strong negative correlation between the two scores. As the symptom scores increased the QALD score decreased and vice versa. It is impossible for changes in these local and general AEs to be quantified in the weekly-administered questionnaire as the scores have returned to or gone above baseline values after only three days. This provides a strong use case for daily SF36v2 questionnaires and a valid case to adopt QALD as a quantifiable measure.

### Limitations of the study

Due to the difficulties in recruitment, the number of participants was low, and the pre-planned age stratification was not possible as there was only one participant older than 64 years of age. The study design called for the administration of a high rate of patient-recorded diary cards and questionnaires including a period of seven consecutive days. While this was necessary to help begin validation of the daily questionnaire in this context, it may cause ‘fatigue’ of completing the questionnaire accurately over a seven-day period. For example, as the patients become more familiar with the questions, pre-empted answers or a lack of consideration or motivation when completing the questions may bias the outcome. Recalibration of the questionnaire is perhaps needed to stop a potential response shift phenomenon specific to the short-term use of patient-recorded questionnaires [15].

### Future research and considerations

Understanding the safety and reactogenicity of any newly developed vaccine, regardless of the absence of severe AEs. As with the majority of adjuvanted vaccines, the AS03-adjuvanted H5N1 has a short-term reactogenicity during the first three days after vaccination. It is important to understand how the reactogenicity affects quality of life of people who have been administered with an adjuvanted vaccine. The discrete changes in QALD-scores reported in this study offer an insight needed to understand the impact of associated symptoms adequately. Developing a predetermined threshold to describe the clinical significance of changes in QALD scores may help to establish a method to allow direct comparisons with other products. Strangfeld et al. (2009) [16], for example established a predetermined hazard ratio threshold of 2.5 to measure clinical significance. As a potential future study consideration may be to use a questionnaire that has a reduced question number, for example, the SF-12 and SF-20 have only 12 and 20 questions respectively and are comparable to the SF-36 regarding reliability and sensitivity [17].

## Conclusion

In conclusion, no safety concerns were associated with the AS03- adjuvanted H5N1 vaccine, synonymous with other large H5N1 trials. These results help to validate our initial development of the novel use of the SF-36v2 questionnaire that has now been shown capable of capturing sensitive changes in average QALD scores over short periods of time. These results suggest that the SF-36v2 can capture that effect, something that the utility instrument with weekly recalls cannot measure. We believe that this novel yet straightforward innovation has the potential to understand the reactogenicity during the development of new vaccines more efficiently

## Additional files

Additional file 1: Relevance, Aims and main Findings of the Study. (PDF 90 kb)

Additional file 2: **Table S1.** All Participants: Changes from baseline (Day 0) for SF-36v2 weekly questionnaire subscale component, summary component and QALD scores at Days 6, 21 and 27 (Total Vaccinated Cohort). **Table S2.** All participants: Changes from baseline (pre dose 1 Day 0) in the SF-36v2 daily questionnaire subscale component, component summary, and QALD scores (Total Vaccinated Cohort). (DOCX 60 kb)

## Abbreviations

AE: Adverse event
ATP: According to the protocol
CI: Confidence interval
MAE: Medically-attended adverse event
MCS: Mental component scores
MedDRA: Medical Dictionary for Regulatory Activities
PCS: Physical component scores
pIMD: potential immune-mediated disease
QALD: Quality-adjusted life-day
QALY: Quality-adjusted life-year
QoL: Quality of life
SAE: Serious adverse event
