# Supplementary material for: Usability of daily SF36 questionnaires to capture the QALD variation experienced after vaccination with AS03A-adjuvanted monovalent influenza A (H5N1) vaccine in a safety and tolerability study
Source: Health Qual Life Outcomes. 2019 May 6;17:80. doi: 10.1186/s12955-019-1147-4 (PMC6501410; doi:10.1186/s12955-019-1147-4)
Supplement: Supplementary file 1 — Relevance, Aims and main Findings of the Study. (PDF 90 kb) [file 12955_2019_1147_MOESM1_ESM.pdf]

Focus on the Patient

What is the context?

By increasing the immunogenicity of vaccines, adjuvantation reduces the amount of antigen required to induce immune response, and addresses the vaccine supply challenge in case of pandemic scenario. However, increased reactogenicity is associated with higher immunogenicity vaccine, and the potential impact on the quality of life of an individual receiving an adjuvanted vaccine expressed as a measure over time in quality-adjusted life-years score loss has never been reported..

What is new?

Most reactogenicity occurs within a few days post-vaccination, therefore a novel and sensitive quality of life instrument that capture daily changes was necessary. The SF-36v2 health survey was used on a daily and weekly basis, and the scores were compared with base-line values and with reactogenicity data to evaluate the potential of this typically weekly-administered instrument to a more sensitive period.

What is the impact?

A novel use of the SF36v2 questionnaire has been established showing to be sensitive in capturing daily changes in quality of life.
