# Supplementary material for: Usability of daily SF36 questionnaires to capture the QALD variation experienced after vaccination with AS03A-adjuvanted monovalent influenza A (H5N1) vaccine in a safety and tolerability study
Source: Health Qual Life Outcomes. 2019 May 6;17:80. doi: 10.1186/s12955-019-1147-4 (PMC6501410; doi:10.1186/s12955-019-1147-4)
Supplement: Supplementary file 2 — Table S1. All Participants: Changes from baseline (Day 0) for SF-36v2 weekly questionnaire subscale component, summary component and QALD scores at Days 6, 21 and 27 (Total Vaccinated Cohort). Table S2. All participants: Changes from baseline (pre dose 1 Day 0) in the SF-36v2 daily questionnaire subscale component, component summary, and QALD scores (Total Vaccinated Cohort). (DOCX 60 kb) [file 12955_2019_1147_MOESM2_ESM.docx]

SUPPLEMENTARY tables

Table S1: All Participants: Changes from baseline (Day 0) for SF-36v2 weekly questionnaire subscale component, summary component and QALD scores at Days 6, 21 and 27 (Total Vaccinated Cohort)

| Weekly questionnaire – All participants | | | | | | |
| --- | --- | --- | --- | --- | --- | --- |
|  | | | Day 6 | Day 21 | Day 27 |  |
| Changes from baseline | | Parameters | Value | Value | Value |  |
| Subscale component scores | Physical function | N | 49 | 48 | 49 |  |
|  |  | Mean | 0.905 | 0.803 | 0.944 |  |
|  |  | SD | 4.4191 | 4.5835 | 4.4889 |  |
|  |  | Median | 0.000 | 0.000 | 0.000 |  |
|  |  | Minimum | -1.93 | -7.71 | -5.78 |  |
|  |  | Maximum | 28.92 | 28.92 | 28.92 |  |
|  | Physical health-related role limitations | N | 50 | 49 | 50 |  |
|  |  | Mean | -0.837 | 0.000 | -0.660 |  |
|  |  | SD | 4.4671 | 3.0803 | 4.2713 |  |
|  |  | Median | 0.000 | 0.000 | 0.000 |  |
|  |  | Minimum | -17.61 | -15.41 | -17.61 |  |
|  |  | Maximum | 8.81 | 8.81 | 8.81 |  |
|  | Bodily pain | N | 49 | 48 | 49 |  |
|  |  | Mean | - 0.339 | 1.891 | 0.290 |  |
|  |  | SD | 7.9794 | 7.2026 | 5.9903 |  |
|  |  | Median | 0.000 | 0.000 | 0.000 |  |
|  |  | Minimum | -26.85 | -15.00 | -11.05 |  |
|  |  | Maximum | 19.34 | 23.29 | 15.00 |  |
|  | General health perceptions | N | 50 | 49 | 50 |  |
|  |  | Mean | -0.596 | -0.729 | -0.776 |  |
|  |  | SD | 3.2414 | 3.7846 | 3.9615 |  |
|  |  | Median | 0.000 | 0.000 | 0.000 |  |
|  |  | Minimum | -9.93 | -15.44 | -15.44 |  |
|  |  | Maximum | 7.50 | 7.50 | 8.82 |  |
|  | Vitality | N | 50 | 49 | 50 |  |
|  |  | Mean | 0.618 | 1.556 | 1.089 |  |
|  |  | SD | 6.5501 | 5.1821 | 5.6351 |  |
|  |  | Median | 0.000 | 2.720 | 0.000 |  |
|  |  | Minimum | -21.77 | -10.89 | -13.61 |  |
|  |  | Maximum | 19.05 | 13.61 | 16.33 |  |
|  | Social function | N | 49 | 48 | 49 |  |
|  |  | Mean | -0.202 | 0.515 | 0.202 |  |
|  |  | SD | 5.6147 | 5.6147 | 5.3363 |  |
|  |  | Median | 0.000 | 0.000 | 0.000 |  |
|  |  | Minimum | -19.77 | -9.89 | -19.77 |  |
|  |  | Maximum | 14.83 | 9.89 | 9.89 |  |
|  | Emotional health-related role function | N | 50 | 49 | 50 |  |
|  |  | Mean | -0.535 | 0.078 | 0.381 |  |
|  |  | SD | 5.3988 | 4.2307 | 3.7979 |  |
|  |  | Median | 0.000 | 0.000 | 0.000 |  |
|  |  | Minimum | -22.90 | -22.90 | -11.45 |  |
|  |  | Maximum | 11.45 | 11.45 | 11.45 |  |
|  | Mental health | N | 50 | 49 | 50 |  |
|  |  | Mean | 0.446 | 0.757 | -0.198 |  |
|  |  | SD | 0.446 | 0.757 | 5.1011 |  |
|  |  | Median | 0.000 | 0.000 | 0.000 |  |
|  |  | Minimum | -14.87 | -9.91 | -17.35 |  |
|  |  | Maximum | 7.43 | 7.44 | 7.44 |  |
| Summary component scores | PCS | N | 49 | 48 | 49 |  |
|  |  | Mean | -0.071 | 0.660 | 0.205 |  |
|  |  | SD | 4.1313 | 3.7903 | 3.5668 |  |
|  |  | Median | 0.000 | 0.180 | -0.290 |  |
|  |  | Minimum | -11.19 | -11.32 | -6.36 |  |
|  |  | Maximum | 13.58 | 13.58 | 13.58 |  |
|  | MCS | N | 49 | 48 | 49 |  |
|  |  | Mean | -0.009 | 0.487 | 0.252 |  |
|  |  | SD | 4.1652 | 2.4585 | 3.8570 |  |
|  |  | Median | 0.420 | 0.095 | 0.000 |  |
|  |  | Minimum | -11.08 | -5.10 | -7.29 |  |
|  |  | Maximum | 11.83 | 7.33 | 11.83 |  |
| QALD Scores | QALD | N | 48 | 48 | 48 |  |
|  |  | Mean | 0.017 | 0.02 | 0.017 |  |
|  |  | SD | 0.1098 | 0.0879 | 0.1116 |  |
|  |  | Median | 0.000 | 0.000 | 0.000 |  |
|  |  | Minimum | -0.25 | -0.25 | -0.25 |  |
|  |  | Maximum | 0.33 | 0.33 | 0.33 |  |

N, number of participants in a given category; SD, standard deviation; PCS, physical component scores; MCS, mental component scores; QALD, quality-adjusted life day.

All participants with grade 3 solicited AEs were 18 – 64 years of age.

Dose 1 administered on Day 0 after completion of the QoL evaluation.

Dose 2 administered on Day 21 after completion of the QoL evaluation.

Thus, data pertain to four intervals: 7-day period post dose 1, 21 days post dose 1, 27 days post dose 1, and 7-day period post dose 2.

Table S2: All participants: Changes from baseline (pre dose 1 Day 0) in the SF-36v2 daily questionnaire subscale component, component summary, and QALD scores (Total Vaccinated Cohort)

| Changes (relative to pre-dose 1 Day 0) in daily questionnaire scores after dose 1 – all participants | | | | | | | | |
| --- | --- | --- | --- | --- | --- | --- | --- | --- |
| Parameter | | Day | | | | | | |
|  |  | 0 | 1 | 2 | 3 | 4 | 5 | 6 |
| Subscale component scores | | | | | | | | |
| Physical function | N | 47 | 49 | 48 | 48 | 49 | 49 | 49 |
|  | Mean | -0.2866 | -0.2751 | -0.3619 | -0.4823 | -0.2755 | 0.0394 | 0.4718 |
|  | SD | 5.3019 | 5.3956 | 6.8158 | 6.9177 | 6.6209 | 5.3015 | 4.3913 |
|  | Median | 0.0000 | 0.000 | 0.000 | 0.000 | 0.0000 | 0.0000 | 0.0000 |
|  | Minimum | -17.3500 | -19.2800 | -34.7100 | -34.7100 | -34.7100 | -21.2100 | -7.7100 |
|  | Maximum | 28.9200 | 28.9200 | 28.9200 | 28.9200 | 28.9200 | 28.9200 | 28.9200 |
| Physical health-related role limitations | N | 47 | 48 | 48 | 48 | 49 | 49 | 49 |
|  | Mean | -0.4215 | -0.8256 | -0.6421 | -1.1465 | -0.5392 | -0.1347 | 0.0898 |
|  | SD | 3.7198 | 5.8456 | 6.5650 | 7.3092 | 6.1879 | 4.5698 | 0.0898 |
|  | Median | 0.0000 | 0.000 | 0.000 | 0.000 | 0.0000 | 0.0000 | 0.0000 |
|  | Minimum | -17.6100 | -26.4200 | -30.8200 | -35.2300 | -33.0300 | -17.6100 | -19.8200 |
|  | Maximum | 8.81 | 8.81 | 8.81 | 8.81 | 8.81 | 8.81 | 8.81 |
| Bodily pain | N | 49 | 48 | 48 | 48 | 49 | 49 | 49 |
|  | Mean | -3.5451 | -2.0563 | 0.1227 | 0.0162 | 0.4029 | 1.5390 | 1.8833 |
|  | SD | 7.0608 | 6.4794 | 6.7025 | 6.9684 | 7.6348 | 6.7120 | 5.9983 |
|  | Median | 0.0000 | 0.000 | 0.000 | 0.000 | 0.0000 | 0.0000 | 0.0000 |
|  | Minimum | -30.7900 | -23.2900 | -30.7900 | -30.7900 | -30.7900 | -23.2900 | -15.0000 |
|  | Maximum | 10.2600 | 10.2600 | 15.0000 | 11.0500 | 15.0000 | 15.0000 | 19.3400 |
| General health perception | N | 49 | 49 | 49 | 49 | 49 | 49 | 49 |
|  | Mean | -1.4861 | -1.1257 | -0.9722 | -1.0627 | -1.0084 | -1.0355 | -0.9904 |
|  | SD | 4.5228 | 4.0842 | 4.0522 | 3.7024 | 4.5098 | 3.6569 | 3.7514 |
|  | Median | -1.3200 | 0.000 | 0.000 | 0.000 | 0.0000 | 0.0000 | 0.0000 |
|  | Minimum | -16.7700 | -12.3600 | -13.2400 | -13.2400 | -17.6400 | -12.3600 | -11.0300 |
|  | Maximum | 8.8200 | 8.8200 | 8.8200 | 8.8200 | 8.8200 | 8.8200 | 8.8200 |
| Vitality | N | 49 | 49 | 49 | 49 | 49 | 49 | 48 |
|  | Mean | -1.7214 | -0.3886 | 0.9075 | -0.0551 | 0.7220 | 0.3890 | -0.1127 |
|  | SD | 7.1291 | 6.8943 | 6.0562 | 7.5867 | 7.6331 | 6.9384 | 6.3757 |
|  | Median | 0.000 | 0.000 | 0.000 | 0.000 | 0.000 | 0.000 | 0.000 |
|  | Minimum | -21.7700 | -21.7700 | -19.0600 | -21.7700 | -21.7700 | -24.4900 | -24.4900 |
|  | Maximum | 21.7700 | 21.7700 | 13.6100 | 21.7700 | 21.7700 | 21.7700 | 10.8900 |
| Social function | N | 49 | 49 | 49 | 49 | 49 | 49 | 49 |
|  | Mean | -0.0002 | -0.9084 | -0.7065 | -0.6053 | -0.2018 | 0.4031 | 0.8069 |
|  | SD | 4.3980 | 5.9417 | 7.0629 | 7.3201 | 5.5221 | 3.7529 | 3.9526 |
|  | Median | 0.000 | 0.000 | 0.000 | 0.000 | 0.000 | 0.000 | 0.000 |
|  | Minimum | -19.7700 | -29.6600 | -29.6600 | -29.6600 | -24.7100 | -9.8900 | -4.9500 |
|  | Maximum | 14.8200 | 14.8200 | 19.7600 | 19.7600 | 19.7600 | 19.7600 | 24.7100 |
| Emotional health-related role function | N | 47 | 48 | 48 | 48 | 49 | 49 | 49 |
|  | Mean | -0.2440 | -0.1588 | 0.1590 | -0.3181 | -0.4676 | 0.0778 | 0.1557 |
|  | SD | 2.5673 | 4.7214 | 2.8344 | 5.2726 | 6.3126 | 3.8565 | 3.5676 |
|  | Median | 0.000 | 0.000 | 0.000 | 0.000 | 0.000 | 0.000 | 0.000 |
|  | Minimum | -7.6400 | -22.9000 | -7.6400 | -30.5400 | -38.1700 | -19.0900 | -15.2700 |
|  | Maximum | 11.4500 | 11.4500 | 11.4500 | 11.4500 | 11.4500 | 11.4500 | 11.4500 |
| Mental health | N | 49 | 49 | 48 | 49 | 49 | 49 | 48 |
|  | Mean | -0.1520 | -0.4547 | 0.1027 | -0.2031 | 0.3031 | 0.3022 | -0.1552 |
|  | SD | 4.9146 | 5.5330 | 3.3498 | 4.2567 | 4.3397 | 4.3685 | 4.8055 |
|  | Median | 0.0000 | 0.000 | 0.000 | 0.000 | 0.0000 | 0.0000 | 0.0000 |
|  | Minimum | -22.3000 | -32.2100 | -9.9100 | -12.3900 | -14.8600 | -12.3900 | -19.8200 |
|  | Maximum | 14.8700 | 9.9100 | 7.4300 | 7.4300 | 12.3900 | 7.4300 | 7.4300 |
| PCS | N | 47 | 48 | 47 | 47 | 48 | 49 | 48 |
|  | Mean | -1.8579 | -1.1960 | -0.5696 | -0.7643 | -0.3857 | 0.1251 | 0.5985 |
|  | SD | 4.8649 | 5.2471 | 6.8465 | 6.5949 | 6.0329 | 4.6996 | 4.0169 |
|  | Median | -1.0300 | -0.5100 | 0.0800 | 0.0000 | 0.0700 | 0.0800 | 0.1150 |
|  | Minimum | -22.5200 | -22.3100 | -38.6400 | -33.6200 | -31.2600 | -20.5900 | -13.3900 |
|  | Maximum | 14.8900 | 14.7400 | 13.3300 | 14.0400 | 14.0400 | 14.0400 | 14.0400 |
| MCS | N | 47 | 48 | 47 | 47 | 48 | 49 | 48 |
|  | Mean | -0.2011 | -0.2398 | 0.3753 | -0.1948 | 0.1673 | 0.2561 | -0.1023 |
|  | SD | 3.5470 | 4.1893 | 3.1442 | 3.9154 | 4.1494 | 3.1001 | 3.2305 |
|  | Median | 0.0400 | 0.4700 | 0.4700 | 0.0750 | 0.6000 | 0.4000 | 0.3850 |
|  | Minimum | -14.8500 | -19.3500 | -9.1700 | -13.2200 | -14.2000 | -8.0600 | -9.7000 |
|  | Maximum | 10.7900 | 7.3200 | 9.7200 | 9.7200 | 14.0500 | 9.7200 | 9.7000 |
| QALD | N | 47 | 48 | 47 | 47 | 48 | 49 | 48 |
|  | Mean | -0.0169 | -0.0047 | 0.0085 | 0.0108 | 0.0188 | 0.0193 | 0.0252 |
|  | SD | 0.0957 | 0.1042 | 0.1029 | 0.1134 | 0.1210 | 0.0957 | 0.0884 |
|  | Median | 0.000 | 0.000 | 0.000 | 0.000 | 0.000 | 0.000 | 0.000 |
|  | Minimum | -0.2660 | -0.3570 | -0.4030 | -0.4030 | -0.3900 | -0.3420 | -0.2230 |
|  | Maximum | 0.2200 | 0.2550 | 0.2490 | 0.2910 | 0.2910 | 0.2910 | 0.2910 |

N, number of participants; SD, standard deviation; PCS, physical component scores; MCS, mental component scores; QALD, quality-adjusted life day
